# Therapeutic approaches for liver fibrosis/cirrhosis by targeting pyroptosis

**DOI:** 10.1515/biol-2025-1092

**Published:** 2025-07-11

**Authors:** Guanen Qiao, Pan Li, Meng Wang, Changjuan Li, Yanmei Wang, Shuanli Xin, Haitao Liu

**Affiliations:** Department of Gastroenterology, First Hospital of Handan City, No. 24 on Congtai Road, Handan, Hebei, 056002, China; Department of Surgery, Handan Infectious Disease Hospital, Handan, Hebei, 056000, China; Department of Internal Medicine, First Hospital of Handan City, Handan, Hebei, 056002, China

**Keywords:** liver fibrosis/cirrhosis, pyroptosis, inhibitors of pyroptosis pathway, natural plant compounds, biomaterials

## Abstract

Long-term repeated exposure to various stimuli leads to chronic liver damage, inflammation, fibrosis, and eventually cirrhosis. Pyroptosis, a mode of inflammatory programed cell death, affects the progression of liver fibrosis/cirrhosis. However, current research on drugs targeting the pyroptosis pathway as a therapeutic strategy in liver fibrosis and cirrhosis remains limited. This review aims to explain the relationship between pyroptosis and liver cirrhosis and focuses on methods for the treatment of liver cirrhosis based on targeted pyroptosis. Here, 31 inhibitor drugs that target inflammasomes, gasdermin D, or caspases are discussed. Although the inhibitory effect of these drugs on pyroptosis is indisputable, their efficacy on cirrhosis needs a thorough investigation. Seventeen natural plant compounds that improve liver fibrosis/cirrhosis in cellular and animal models through targeting pyroptosis are also reviewed. In addition, stem cell replacement therapy and exosomes have broad application prospects in liver cirrhosis from the perspective of pyroptosis. In the future, the primary challenges will involve validating the efficacy of the aforementioned drugs in targeting pyroptosis for cirrhosis treatment using human liver organoids as well as determining their potential clinical application.

## 1 Introduction

Cirrhosis is widely prevalent with a high mortality rate. It is the third leading cause of death in people aged 45–64 years and has become one of the major health problems worldwide [1]. Cirrhosis is the major predisposing risk factor to hepatoma carcinoma and is a consequence of end-stage liver disease, which results from long-term repeated exposure to various stimuli leading to chronic liver damage, inflammation, fibrosis, and eventually cirrhosis [2]. The etiology of cirrhosis mainly includes viral infection (such as hepatitis B virus [HBV], hepatitis C virus [HCV], and hepatitis D virus, either alone or superimposed on HBV); alcohol intake; metabolic and genetic, autoimmune, biliary, vascular, drug-induced (such as vitamin A, amiodarone, methotrexate, and methyldopa), and cholestatic diseases; and cryptogenic cirrhosis (with uncertain cause) [3,4]. The development of cirrhosis involves multiple cell types, such as hepatocytes, hepatic stellate cells (HSCs), Kupffer cells, and liver sinusoidal endothelial cells. In the treatment of cirrhosis, the primary objective is to address the disease cause as effectively as possible and delay hepatic decompensation because mitigating the cause increases the likelihood of recovery [5]. Despite the complexity and diversity of cirrhosis, promising advances in cirrhosis treatment remain elusive, and to date no treatment with sustained effectiveness has been approved for liver fibrosis. In addition, the unclear pathogenesis of cirrhosis greatly limits the development and application of targeted drugs. Therefore, the pathomechanisms of cirrhosis must be elucidated to facilitate the development of its therapeutic strategies and improve patient benefits.

Pyroptosis refers to inflammatory programed cell death mediated by inflammasomes that are controlled by classical and nonclassical signaling pathways [6]. In general, inflammasomes trigger the activation of caspases, which leads to the polymerization and cleavage of gasdermin (GSDM) family members, resulting in cell perforation and release of inflammatory factors. Pyroptosis can detect danger signals, such as persistent cell swelling until lysis, resulting in the release of cell contents and triggering of a strong inflammatory response. A distinctive feature of pyroptosis is that cell death is accompanied by the release of numerous inflammatory factors such as interleukin (IL)-18 and IL-1β. Thus, pyroptosis is an important innate immune program of the body and takes on a crucial role in disease resistance and fighting infection. In recent years, numerous studies have suggested that pyroptosis is closely related to various liver diseases, such as cirrhosis, non-alcoholic fatty liver disease (NAFLD), and alcoholic liver disease (ALD) [7,8]. Therefore, targeted regulation of the cellular pyroptosis pathway may be a potential strategy for the treatment of cirrhosis. For example, a study demonstrated that the inhibition of pyroptosis by targeting caspase-1 or NOD (nucleotide-binding and oligomerization domain)-like receptor (NLR) protein 3 (NLRP3) significantly inhibits liver fibrosis or cirrhosis [9]. Therefore, summarizing and predicting the methods and targets that can be utilized for the treatment of cirrhosis based on pyroptosis are clinically important.

Overall, pyroptosis has not been studied much in cirrhosis compared with other diseases, and there are insufficient articles on its relevance. In this review, we summarize the mechanism of pyroptosis and pyroptosis-based approaches for cirrhosis, including inhibitors of the pyroptosis pathway, natural plant compounds, and other effective approaches. This information may provide ideas for research on the treatment of cirrhosis.

## 2 Pyroptosis mechanism

### 2.1 Canonical pathway of pyroptosis

**Figure 1:**
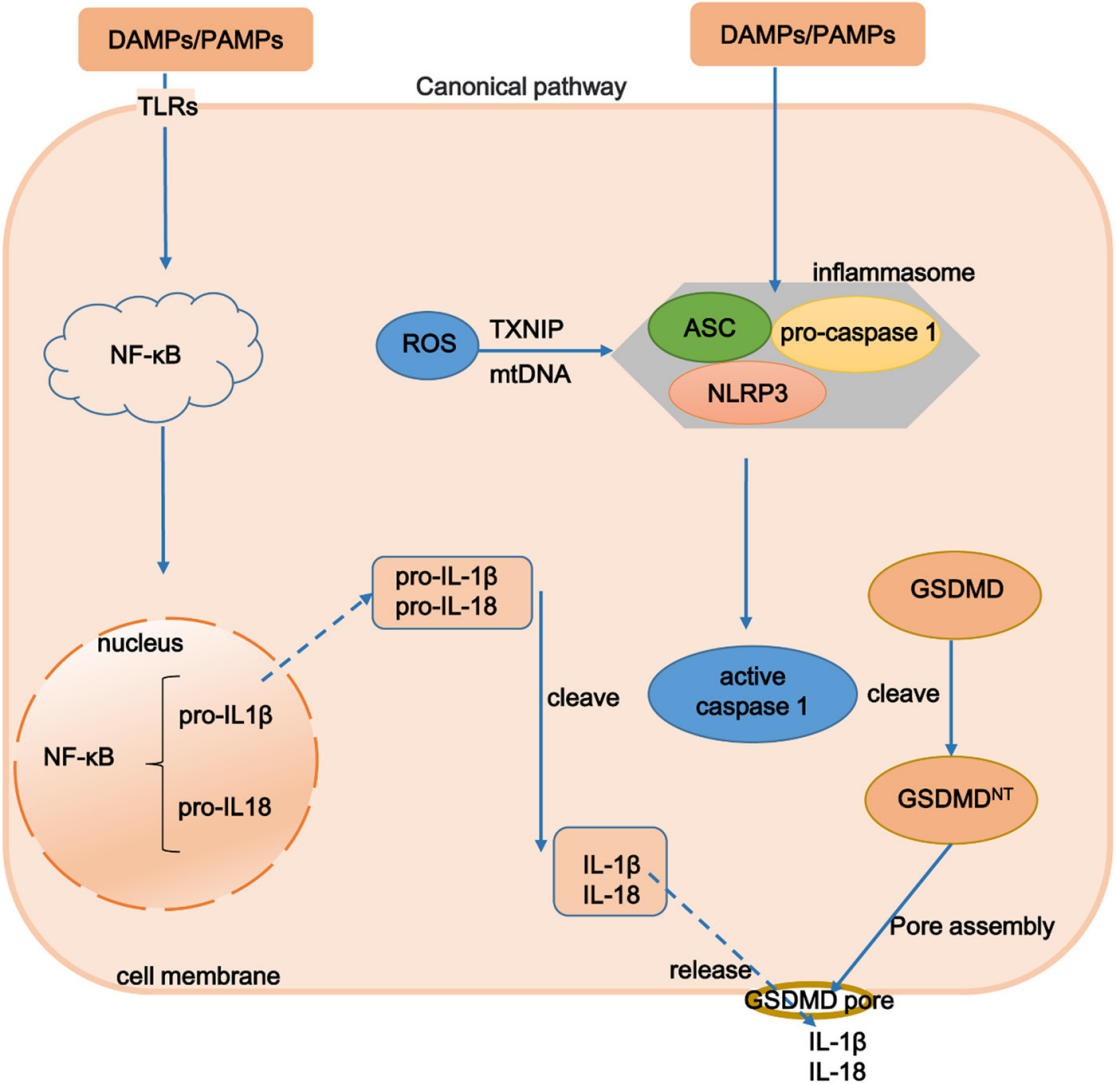
Canonical pathway of pyroptosis. In the canonical pathway, inflammasomes are regulated by PRRs, including various DAMPs, PAMPs, and ROS, to activate caspase-1 and then cleaved the executor protein GSDMD into GSDMD-N fragments that form “pores” on the cell membrane to allow for the release of proinflammatory cytokines such as interleukin (IL)-1β and IL-18. PRRs, pattern recognition receptors; DAMPs, damage-associated molecular patterns; GSDMD, gasdermin D; PAMPs, pathogen-associated molecular patterns; ROS, reactive oxygen species.

In the canonical pathway, inflammasomes can be regulated by pattern recognition receptors (PRRs), such as damage-associated molecular patterns (DAMPs) and pathogen-associated molecular patterns (PAMPs), to activate IL-1β and IL-18 to generate and release proinflammatory cytokines [10]. The canonical pathway involves two signaling pathways. First, DAMPs and PAMPs are recognized by PRRs on the cell membrane surface, which triggers the downstream MyD88-NF-κB signaling pathway to produce pro-IL-1β and pro-IL-18 (Figure 1) [11]. Second, caspase-1 is the only caspase that exists in the canonical pathway and can be activated by inflammasomes [12]. As shown in Figure 1, DAMPs and PAMPs enter cells and activate the inflammasomes to form multiprotein complexes and subsequently activate caspase-1. Then, active caspase-1 cleaved the executor protein GSDMD into 22 and 31 kDa active N-terminal and C-terminal fragments. GSDMD-N fragments cleave phosphatidylinositol/cardiolipid-containing liposomes from cell membranes and “punch” holes in the cell membrane [13]. Meanwhile, caspase-1 completes the cleavage and maturation of pro-IL-1β and pro-IL-18 to form active proinflammatory factors IL-1β and IL-18 [14]. IL-1β and IL-18 are secreted outside the cell through the “pores” formed by GSDMD-N in the cell membrane, inducing an inflammatory response called pyroptosis [10,13].

In addition, an imbalance between mitochondrial homeostasis and reactive oxygen species (ROS) production is associated with pyroptosis. ROS are mainly produced by mitochondria and mediate various mitochondrial dysfunctions. Nicotinamide adenine dinucleotide phosphate oxidase 2 (NOX2)-derived ROS can activate NLRs in NLRP3 inflammasomes in macrophages [15,16]; however, whether NOX2 is involved in NLRP3 activation is controversial. Currently, two main pathways of ROS-induced cellular pyroptosis are known (Figure 1). First, mitochondrial ROS (mtROS) inhibit the synthesis of related protein subunits responsible for encoding mtDNA, potentially disrupting mitochondrial oxidative phosphorylation, releasing more mtROS, and continuously damaging mtDNA, creating a vicious cycle of bioenergetic disruption and ultimately leading to pyroptosis [16]. Second, with the increase in ROS, thioredoxin separates from thioredoxin-interacting protein (TXNIP) and binds to NLRP3, leading to NLRP3 activation [17].

### 2.2 Non-canonical pathway

**Figure 2:**
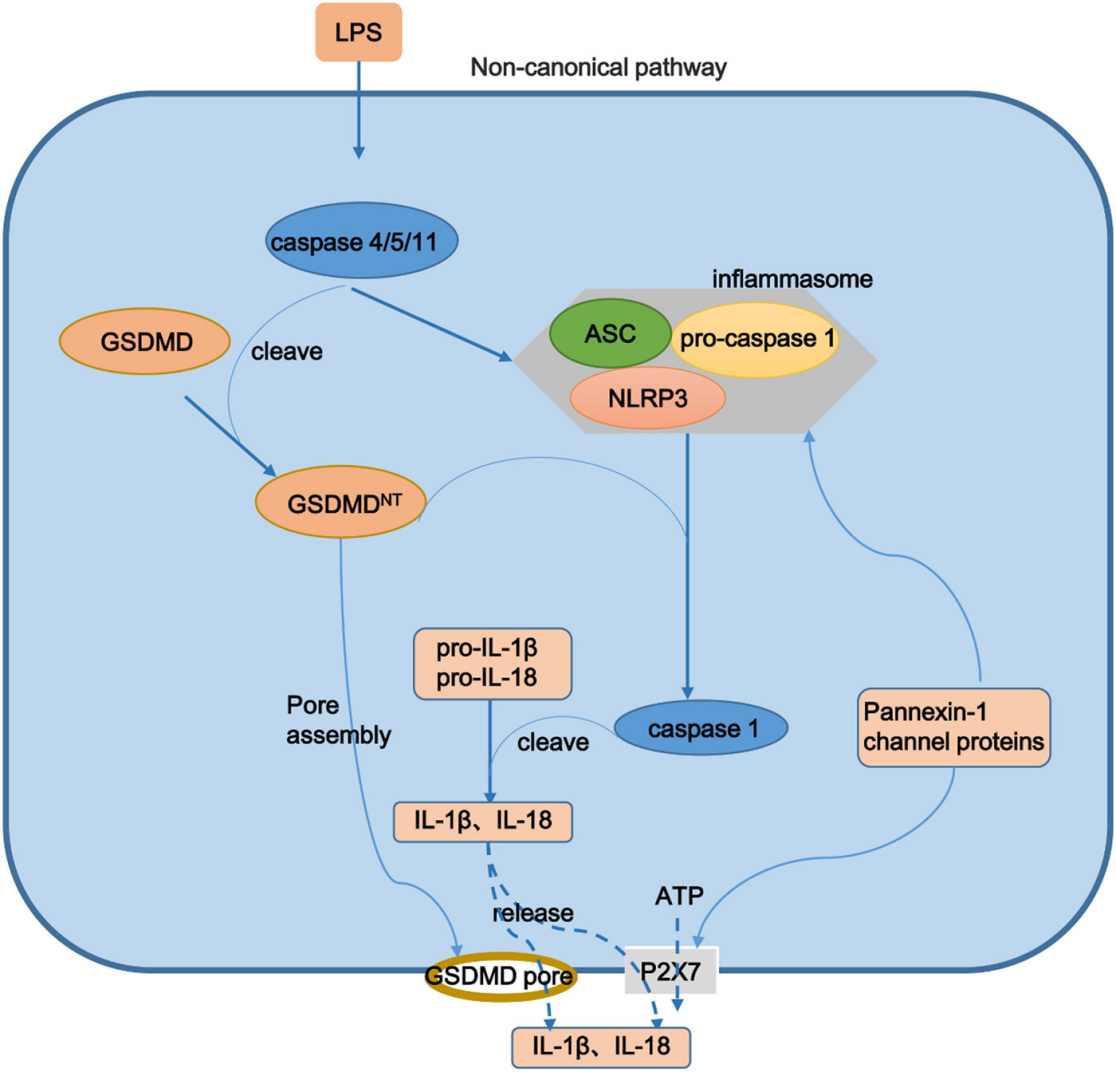
Non-canonical pathway of pyroptosis. In the non-canonical pathway, LPS binds to caspases 4 and 5 (human) or caspase-11 (mouse) and initiates pyroptosis, which is very similar to that induced via the canonical pathway. LPS, lipopolysaccharide.

Unlike caspase-1 dependence in the classical pathway, in the non-canonical pathway, other PRRs such as lipopolysaccharide (LPS) can directly bind to caspases 4, 5, and 11 to induce pyroptosis (Figure 2). Notably, caspases 4, 5, and 11 cannot cleave pro-IL-1β/pro-IL-18 but can cleave the GSDMD [18]. Caspase-11 is mainly present in mice, whereas caspases 4 and 5 are mainly present in humans [19]. Activated caspases 4, 5, and 11 cleave GSDMD to biologically active GSDMD-NT, which further activates the downstream factor caspase-1 by binding to NLRP3 inflammasomes and ultimately secretes mature IL-1β and IL-18 outside cells [20,21]. As shown in Figure 2, Pannexin-1 channel proteins can open P2X7 cell membrane channels, release ATP under the action of caspase-11, and then form “holes” in the cell membrane, inducing pyroptosis [22]. Moreover, the activation of pannexin-1 channel protein promotes the release of intracellular K^+^ ions and activates NLRP3, which also promotes the release of IL-1β and IL-18 [23].

### 2.3 Other mechanism pathways of pyroptosis

Some studies have confirmed some mechanisms of focal death independent of classical and nonclassical mechanisms; however, the mechanisms are not fully defined yet. In 2020, granzyme B was found to activate caspase-3 to cleave gasdermin family protein E (GSDME), resulting in extensive pyroptosis [24]. Moreover, granzyme A was found to cleave the GSDMB at non-aspartic acid sites, which causes pyroptosis [25]. Thus, the granzyme-mediated pathway is another mechanism of pyroptosis. Second, recent studies have shown that GSDME plays a role in mediating pyroptosis [26]. Unlike GSDMD, GSDME can be cleaved at specific sites by caspase-3, a key factor in apoptosis, and the cleaved GSDME-N fragment can penetrate cell membranes. Caspase-3 is activated by tumor necrosis factor-α and induces apoptosis; however, if cells can express GSDME, the mode of cell death shifts rapidly to pyroptosis or even direct pyroptosis [26]. Notably, the addition of caspase inhibitors to cells or GSDME knockdown will prevent pyroptosis [27]. *In vivo* tests showed the same effect, with chemotherapeutic drugs producing significantly fewer side effects in GSDME-knockout mice [28]. Thus, the GSDME-mediated pathway is another mechanism of pyroptosis. Third, the caspase-3/8-mediated pathway may also play a role in pyroptosis. Although it was previously thought that apoptosis-related caspases (e.g., caspases 3 and 8) could not stimulate GSDMD-induced pyroptosis, recent studies have found that chemotherapeutic agents can induce caspase-3-mediated cleavage of highly expressed GSDME, leading to tumor cell pyroptosis [27,28]. In addition, the YopJ protein can suppress transforming growth factor-beta (TGF-β)-activated kinase 1 and induce caspase-8-related cleavage of GSDMD, resulting in pyroptosis [29,30]. Thus, another pathway is the caspase-3- or caspase-8-mediated pyroptosis pathway. Interestingly, caspase-3 is generally considered one of the important markers for apoptosis, and this finding links two regulated cell death processes, namely, apoptosis and pyroptosis, and is important to further explore the mechanism of pyroptosis.

## 3 Pyroptosis and liver cirrhosis

**Figure 3:**
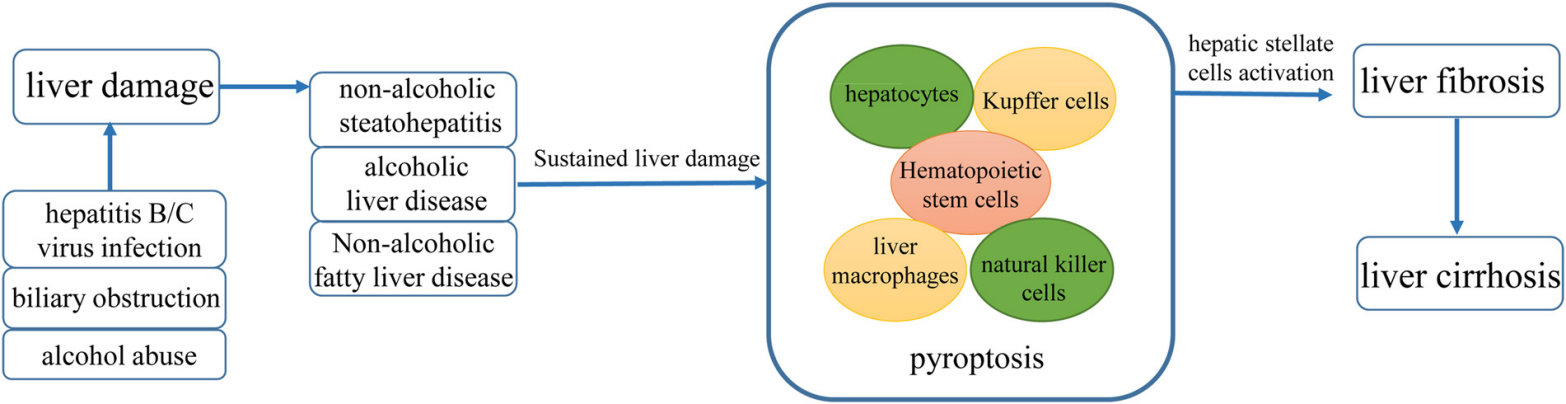
Relationship between pyroptosis and cirrhosis. HBV or HCV infection, biliary obstruction, and alcohol abuse could cause liver damage, which progresses to different liver diseases. In the early stages of liver disease-related inflammation, the activation of inflammasomes in hepatocytes and Kupffer cells induces the release of a plethora of proinflammatory compounds, which will activate HSCs and evoke a state of fibrosis, degenerating to end-stage cirrhosis.

As shown in Figure 3, cirrhosis is the final stage of several liver diseases. Liver fibrosis is a prevalent pathological process associated with various chronic liver diseases, which is primarily driven by hepatocellular damage [3]. Liver fibrosis is characterized by the activation and transformation of HSCs into myofibroblasts, which synthesize collagen fibers. This process results in the progressive accumulation of the extracellular matrix, fibrous scar formation, and disruption of the physiological structure of the liver. Increasing studies have shown that pyroptosis is a strong trigger for HSC activation, fiber formation, fibrosis, and even cirrhosis. For example, in clinical studies, patients with cirrhosis have shown elevated levels of circulating GSDMD, IL-1β, and IL-18 [31]. Excessive activation of NLRP3 inflammasomes in mice can lead to hepatocyte pyroptosis and cause severe liver inflammation and fibrosis, and pyroptosis products IL-1β and IL-18 regulate the activation of HSCs and promote liver fibrosis *in vitro* [32]. NLRP3 inflammasome activation is required for liver inflammation and fibrosis in mouse models of NAFLD and non-alcoholic steatohepatitis (NASH) [33]. Moreover, pyroptosis of various cells is crucial in cirrhosis development. For example, pyroptosis of hepatocytes and release of inflammasome particles stimulate the activation of HSCs, leading to liver fibrosis [8]. Ursolic acid mitigates Kupffer cell pyroptosis in liver fibrosis by modulating the NOX2/NLRP3 inflammasome signaling pathway [34]. The activation of inflammasomes in natural killer (NK) cells promotes HSC apoptosis and mitigates the progression of liver fibrosis via tumor necrosis factor-related apoptosis-inducing ligand (TRAIL)-dependent degranulation [35]. Soluble egg antigens of *Schistosoma japonicum* trigger HSC pyroptosis, thereby intensifying liver damage [36]. In summary, pyroptosis is an important cause of liver fibrosis and even cirrhosis.

## 4 Pyroptosis markers that can be used as drug targets

To date, no treatment with sustained effectiveness has been approved for the management of liver fibrosis, highlighting the need to explore specific therapeutic targets. Different forms of pyroptosis are closely associated with cirrhosis. Researchers are currently trying to improve the therapeutic efficacy of liver fibrosis/cirrhosis through pyroptosis. Pyroptosis can be primarily inhibited in two main ways: by blocking the activation of NLRP3 inflammasomes and inhibiting the GSDM family signaling pathway. Herein, we summarize the role of inhibitors of the pyroptosis pathway, natural plant compounds, and other effective approaches in the anti-pyroptosis process to provide data for the development of clinical treatment of liver fibrosis/cirrhosis.

### 4.1 Inflammasome-related therapy

Inflammasomes, multiprotein complexes, are an important component of pyroptosis, which are composed of a sensor molecule, apoptosis-associated speck-like protein (ASC), and pro-caspase-1 (Figure 1) [37]. Inflammasomes can be divided into two types according to their receptors: NLR family (NLRP1/2/3/6, NLRC4, and NLRP12) and the pyrin and hematopoietic interferon-inducible nuclear protein (HIN) domain family including absent in melanoma 2 (AIM2) and pyrin [38]. Inflammasomes play a crucial role in recognizing cellular stress to activate caspases that induce pyroptosis [39]. NLRC4 depletion provides protection against hepatocyte pyroptosis in mice [40]. Therefore, inflammasome inhibition may be a promising treatment strategy in liver fibrosis/cirrhosis.

A large number of small molecule inhibitors can be targeted to inhibit the activation of NLRP3 inflammasomes, as shown in Table 1. MCC950, originally known as a cytokine release inhibitor, specifically inhibits NLRP3 inflammasome activity in canonical and non-canonical pathways [41]. In a mouse model of cholestatic liver injury, MCC950 application significantly reduces the production of proinflammatory cytokines and incidence of hepatocyte death and attenuated cholestatic liver injury and liver fibrosis by inhibiting NLRP3 activation and assembly [42]. Compound 59, which targets NLRP3/AIM2-IN-3, can inhibit NLRP3/AIM2 to form inflammasomes that block pyroptosis [43]. P2X7 activation triggers NLRP3 inflammasomes. A438079, a specific inhibitor of P2X7 that acts as a trigger for NLRP3 inflammasomes, attenuates CCL4-induced hepatic fibrosis in mice by downregulating smooth muscle α-actin and TGF-β1 expression and reducing hepatic collagen formation [23]. These studies suggest that small-molecule inhibitors targeting NLRP3 inflammasomes (Table 1) may have the potential to improve cirrhosis. However, the lack of animal and clinical trials has hindered the understanding of the efficacy of these inhibitors in patients with cirrhosis.

**Table 1:** Inhibitor drugs for pyroptosis

Drug	Target
NLRP3/AIM2-IN-3 (compound 59)	NLRP3/AIM2
MCC950	NLRP3
A438079	P2X7/NLRP3
CY-09	NLRP3
JC2-11	Inflammasomes
NLRP3-IN-13	NLRP3
NLRP3-IN-9	NLRP3
Dapansutrile	NLRP3
Stavudine (d4T)	NLRP3
Muscone	NF-κB/NLRP3
Emlenoflast (MCC7840) sodium	NLRP3 inflammasomes
Licochalcone B	NEK7‐NLRP3
Ruscogenin	TXNIP/NLRP3
Arglabin ((+)-Arglabin)	NLRP3 inflammasomes
CORM-3	NLRP3 inflammasomes
YQ128	NLRP3
Soyasaponin II	YB-1 and NLRP3
NLRP3-IN-10	NLRP3
NLRP3-IN-NBC6	NLRP3
INF4E	NLRP3
Emlenoflast (MCC7840)	NLRP3 inflammasomes
Disulfiram (tetraethylthiuram disulfide)	ALDH1/GSDMD
LDC7559	GSDMD
Ac-FLTD-CMK	GSDMD
2-Bromohexadecanoic acid (2-bromopalmitic acid)	BAK/BAX-baspase 3-GSDME
Ac-FEID-CMK TFA	GSDMEb, caspy2
Ac-FEID-CMK	GSDMEb, caspy2
Pep19-2.5	PRRs
Vx-166	Caspases
Emricasan	Caspases
Ac-YVAD	Caspases

Most of the drugs shown in Table 1 are known to inhibit pyroptosis and thus demonstrate therapeutic effects in various diseases. However, the effectiveness of only a few inhibitors in liver cirrhosis is supported by the literature.

### 4.2 Caspase-related therapy

The caspase family is a class of cysteine proteolytic enzymes that recognize the tetrapeptide sequence of the substrate and cleat it after aspartic acid (D) residues to activate the substrate protein [44]. In the absence of upstream signals, the caspase family remains in the cytoplasm as inactive proenzymes. Substrate recognition and cleavage can only occur when the caspase undergoes self-cleavage and is activated in response to an upstream signal. Currently, a total of 13 caspases have been discovered, and caspases 1, 4, 5, 11, and 12 are related to the pyroptosis signaling pathway [37]. Among them, GSDMD can be cleaved by caspases 1, 4, and 5 in humans and caspase 11 in mice [45]. Some pyroptosis inhibitors based on caspase family members have also been developed. For instance, VX-166 and Emricasan, as caspase inhibitors, show beneficial effects on liver inflammation and fibrosis in NASH mice by decreasing IL-1β and IL-18 expression and inactivating HSCs [46,47]. Ac-YVAD, a caspase-1 specific inhibitor, could block hepatic steatosis and fibrosis in high-fat-diet-fed mice [48]. Caspase-11 deficiency ameliorates liver inflammation and fibrosis by inducing hepatocyte pyroptosis in NALFD mice in *in vitro* experiments, but no specific inhibitor of caspase-11 is available [49]. Although these inhibitors have shown good results in experimental models, it remains unclear whether these inhibitors can improve liver inflammation and fibrosis in patients with cirrhosis in clinical treatment.

### 4.3 Executioner protein GSDM-related therapy

In 2015, GSDMD was confirmed as the executor of pyroptosis [20,50]. The N-terminal of cleaved GSDMD inserts into the plasma membrane of cells and forms stable pores, which enables the release of proinflammatory factors and plays an important role in pyroptosis [13,51]. Butyrate-led inhibition of GSDMD-mediated pyroptosis could ameliorate AFLD [52]. In humans, six genes are related to the GSDM family, namely, *GSDMA*, *GSDMB*, *GSDMC*, *GSDMD*, *GSDME* (also known as *DFNA5*), and *PJVK* (also known as *DFNB59*) [53]. GSDMD cleavage is the most studied process, and GSDMD inhibitor is also currently the most developed inhibitor (Table 1), including disulfiram, LDC7559, Ac-FLTD-CMK, Ac-FEID-CMK, and Ac-FEID-CMK TFA. However, the efficacy of these inhibitors in liver fibrosis/cirrhosis remains to be determined. For example, disulfiram, a dithiocarbamate family member, is widely used in clinical practice as an anti-alcoholic drug, which can inhibit GSDMD expression by covalently modifying cys192 of GSDMD, thereby inhibiting plasma membrane pore formation and pyroptosis [54]. Disulfiram can also inhibit NLRP3 activity and IL-1β production in the downstream pathway by reducing ROS production [55]. However, despite the multitudes of studies on GSDMD, no studies on small-molecule drugs targeting GSDMD for the treatment of cirrhosis can be retrieved from PubMed.

### 4.4 Therapeutic effects of natural plant compounds based on pyroptosis

Traditional Chinese medicines are based on the combination of natural plants, animals, or minerals that are characterized by “multicomponent and multitarget” nature, and their effectiveness in treating various liver diseases has been proven. Although the exact single drug target cannot be known because of the complexity of herbal formulations, emerging studies have demonstrated that pyroptosis is an active target of natural medicines for the treatment of fibrosis/cirrhosis (Table 2).

**Table 2:** Therapeutic effects of natural plant compounds based on pyroptosis in liver fibrosis/cirrhosis

Natural plant compounds	Targets
Jiangzhi Ligan Decoction	GSDMD
Quercetin	PGC-1α/NLRP3 inflammasomes
Mangostin	AMPK/NLRP3
Ursolic acid	NOX2/NLRP3 inflammasomes
Berberine	ROS/TXNIP/NLRP3
Dihydromyricetin	GSDMD
Nobiletin	SIRT1
Naringenin	NF-κB/NLRP3
Cannabidiol	NF-κB/NLRP3
Salidroside	TXNIP/NLRP3
Dieckol	NLRP3 inflammasomes
Antcin A	NLRP3 inflammasome assembly
Oleic acid	Endoplasmic reticulum stress/NLRP3-GSDMD
Taurine	Autophagy
Sestrin2	NLRP3
Peroxiredoxin	NLRP3
Curcumin	JAK2/NLRP3

Jiangzhi Ligan Decoction, a classical Chinese herbal formula, has demonstrated therapeutic effects on high-fat-diet-induced NAFLD and is dependent on the inhibition of the GSDMD-mediated canonical/non-canonical pyroptosis pathway [56] or inflammasome signaling pathway [57].

Quercetin is a dietary phytochemical found in a variety of fruits and vegetables. Owing to its ion chelation and iron stabilization, quercetin has the ability to scavenge ROS and reduce lipid peroxidation [58]. PGC-1α, a key mitochondrial biogenesis regulator, modulates NLRP3 inflammasomes and attenuates pyroptosis in ALD [59]. Quercetin reduced hepatocyte pyroptosis in L02 cells by scavenging mitROS and promoting PGC-1α-mediated mitochondrial dynamics to protect against ethanol-induced hepatocyte pyroptosis. Specifically, quercetin reduces NLRP3 inflammasomes, pro-caspase1/ASC, and GSDMD activation, decreases IL-1β and IL-18 secretion from L02 cells, and alleviates ALD hepatocyte pyroptosis [60].

Mangostin, a naturally occurring carbon glycoside molecule, is abundant in the leaves of the tropical medicinal plant mango. Mangiferin significantly reduces serum triglyceride and free fatty acid levels and improves lipid levels in patients who are overweight and hyperlipidemic [61]. Adenosine monophosphate-activated protein kinase (AMPK) is a key regulatory enzyme of hepatic tissue cell energy homeostasis, which is related to hepatocyte glucolipid metabolism and is a potential target for NAFLD therapy. Mangiferin activates AMPK and significantly downregulates NLRP3, caspase-1, and IL-1β expression and inhibits NLRP3 inflammasome activation-mediated GSDMD cleavage and pyroptosis.

Ursolic acid is a natural triterpene that can be extracted from a variety of plants and has a variety of biological functions, such as antioxidant, anti-inflammatory, hepatoprotective, and immunomodulatory [62]. Ursolic acid inhibits KC pyroptosis in liver fibrosis *in vitro* and *in vivo* by suppressing the protein expression of pyroptosis-related indicators (NLRP3, pro-caspase-1, cleaved caspase-1, GSDMD, GSDMD-N, and IL-1β) in the NOX2/NLRP3 inflammasome signaling pathway [34].

Berberine is an isoquinoline alkaloid derived from certain herbs, including yellow lily, and is used for treating diarrhea and intestinal infections [63]. Berberine improves mitochondrial function, attenuates oxidative stress, lowers serum cholesterol levels, and shows strong beneficial effects on NAFLD [64]. Berberine inhibits NLRP3 inflammasome activation and downstream signaling factor expression via the ROS/TXNIP pathway or NF-κB signaling pathway, ultimately inhibiting hepatocyte pyroptosis and attenuating NAFLD and liver fibrosis [64].

Dihydromyricetin is the most abundant natural flavonoid in vine grapes and demonstrates various pharmacological effects [65]. Dihydromyricetin inhibits CCl4-induced pyroptosis by reducing GSDMD and IL-18 levels [66].

Nobiletin, a herb thought to have antioxidant and anti-tumor properties, is a polymethoxyflavonoid present in citrus peel. Nobiletin inhibited NLRP3 inflammasome activation and the expression of its downstream pathway factors by increasing the expression of SIRT1 and effectively protects AML-12 cells from palmitic acid-induced steatotoxicity [67].

Naringenin is a flavonoid with antioxidant, antifibrotic, anti-inflammatory, and anticancer properties and could prevent liver damage caused by different drugs. Naringenin attenuates NAFLD by reducing the expression levels of NF-κB, NLRP3, IL-1β, and IL-18 in the NF-κB/NLRP3 pathway [68].

Cannabidiol is a nonpsychotropic cannabis-derived compound with a wide range of pharmacological properties, including cardioprotective, antioxidant, and anti-inflammatory abilities [69]. Cannabidiol can attenuate NAFLD and NASH by inhibiting the expression of various factors in the NF-κB/NLRP3 pathway [70,71].

Salidroside, a phenylpropanoid glycoside compound, is the main active ingredient of *Rhodiola rosea*, which grows at high altitudes and has various positive biological activities [72]. Treatment with salidroside improves dyslipidemia and inhibits hepatic lipid deposition and attenuates NAFLD by modulating oxidative stress and inhibiting the TXNIP/NLRP3 pathway [73].

Dieckol is a natural phenolic compound found in kelp. It can decrease the expression of NLRP3, ASC, caspase-1, GSDMD, and cleaved GSDMD to improve NAFLD *in vivo* [74].

Antcin A is a steroidal phytochemical and has potent anti-inflammatory and anti-tumor effects. It can bind to NLRP3 and then inhibit NLRP3 inflammasome assembly and activation, suppressing the inflammatory response in mouse liver tissues and reducing lipid deposition and inflammatory factor levels [75].

Oleic acid is a typical monounsaturated fatty acid and is the predominant component in olive oil (70–80%). It inhibits NLRP3/GSDMD expression by suppressing palmitic acid-induced endoplasmic reticulum (ER) stress, ultimately inhibiting HepG2 cell pyroptosis and reducing hepatic lipotoxicity in NASH rats [76].

Taurine is a sulfur-containing beta-amino acid found in free cells in many tissues of humans and animals. Taurine inhibits arsenic-induced inflammation and pyroptosis through the autophagic-CTSB-NLRP3 inflammasomal pathway, ultimately alleviating NASH [77].

Sestrin is a highly conserved family consisting of Sestrin1, Sestrin2, and Sestrin3 in mammals. Sestrin2 deficiency promotes liver ER stress during cholestasis and exacerbates cholestasis-induced liver fibrosis [78]. Cholestasis-induced Sestrin2 attenuates ER stress via the AMPK/mammalian target of rapamycin complex 1 (mTORC1)-dependent pathway. In cholestatic liver injury, Sestrin2 inhibits NLRP3 activation to suppress pyroptosis, benefiting patients [79].

Peroxiredoxin is a family of thiol peroxidases that scavenge peroxides from cells. Among the six mammalian peroxiredoxins, peroxiredoxin 3 is specifically localized in the mitochondria. Peroxiredoxin 3 eliminates large amounts of hydrogen peroxide and is a major target of peroxynitrite in the mitochondria. Notably, peroxiredoxin 3 could reduce hepatotoxicity through inhibiting the NLRP3 inflammasome signaling pathway by targeting mitochondrial ROS to inhibit pyroptosis [80].

Curcumin, a diketone compound, belongs to polyphenol and is isolated from *Curcuma longa*. Cui et al. demonstrated that curcumin could mitigate liver fibrosis in ducks through inhibiting JAK2/NLRP3-mediated pyroptosis [81].

### 4.5 Biomaterials inhibit liver cirrhosis based on pyroptosis

With the development of science and technology, bioengineering materials have played an irreplaceable role in the medical field. In recent years, the application of biomaterials in liver fibrosis based on the pyroptosis pathway has been gradually discovered.

Stem cells (SCs) have the capacity for self-renewal, multispectral differentiation, and immunomodulation [82]. SC replacement therapy has broad application prospects in liver cirrhosis. For example, SCs from human exfoliated deciduous teeth (SHEDs) have angiogenic differentiation and proliferative potential and are resistant to apoptosis. SHEDs were found to attenuate liver fibrosis by differentiating into hepatocyte-like cells *in vivo*, and SHED-derived hepatocyte transplantation mitigates liver fibrosis [83]. Importantly, SHEDs improve liver cirrhosis by inhibiting the pyroptosis pathway through the suppression of NLRP3 inflammasome-activated GSDMD [83]. Human umbilical cord mesenchymal SCs improve acute liver failure by suppressing inflammation, pyroptosis, and apoptosis [84]. Mesenchymal SCs have also been reported to have possible therapeutic potential in cirrhosis through pyroptosis [85]. However, stem cell therapy for cirrhosis has certain limitations. Currently, there are no unified standards to determine which type of SCs to use, the optimal cell dosage, and the delivery route [86]. Furthermore, the long-term effects and potential side effects of SC therapy require more clinical research for validation. These challenges limit the widespread application of SC therapy in the treatment of liver cirrhosis.

Exosomes, vesicles measuring 30–150 nm in diameter, are secreted by cells and contain functional nucleic acids, proteins, lipids, and cytokines. They have emerged as a promising advanced cell-free therapy in various diseases such as fibrosis/cirrhosis [87]. For instance, exosomes derived from bone marrow mesenchymal SCs mitigate liver fibrosis in cirrhosis rat models by inhibiting PCNA/NLRP3/caspase 1/GSDMD-mediated pyroptosis [88]. Milk-derived exosomes encapsulated with forsythiaside A specifically targeting CD44 could attenuate liver fibrosis by NLRP3-mediated pyroptosis [89]. These studies have indicated that pyroptosis-based exosomes are effective in cells and animals; therefore, they have great potential in the treatment of liver fibrosis. Although exosomes show some potential in the treatment of cirrhosis, there are still some limitations [90,91]. First, the distribution and duration of action of exosomes within the body are still not clear, which limits their clinical application. Second, the technical challenges associated with the production, purification, and large-scale application of exosomes are major barriers to their clinical use. Finally, the safety and long-term effects of exosome therapy require further validation through more clinical research. Furthermore, research on exosome-based nanomaterials for the treatment of liver fibrosis is still in the preclinical trial stage. Before clinical application, rational nanomaterials must be designed, and their hepatotoxicity, pharmacokinetics, and targeted metabolic pathways evaluated systematically.

### 4.6 Clinical therapy in liver cirrhosis

In clinical practice, drug development is an arduous process, in which solid preclinical biology and pharmacology of drugs are important factors for clinical success [92]. Liu et al. discovered that Yaq-001 could significantly reduce liver injury in acute-on-chronic liver failure animals, and Yaq-001 has met the safety and tolerability criteria in clinical trials, providing theoretical evidence for the clinical transformation of Yaq-001 in patients with cirrhosis [93]. The treatment of liver cirrhosis involves the administration of various medications, including antiviral, liver-protecting, and antibacterial drugs, and cell therapy [94,95,96]. Wong et al. found that entecavir reduced the risk of hepatic events and death in patients with chronic hepatitis B and liver cirrhosis [97]. Zeng et al. reported that low-dose rifaximin reduced complications and boosted survival in patients with decompensated liver cirrhosis [98]. Brennan et al. confirmed the safety and potential efficacy of autologous macrophage therapy in the treatment of liver cirrhosis through a Phase II randomized controlled trial [99]. Rinella et al. conducted a randomized trial in patients with NASH and compensated cirrhosis, showing that 3 mg of aldafermin significantly reduced enhanced liver fibrosis scores [100]. Currently, research on the clinical application of pyroptosis in liver cirrhosis is still in the developmental stage; however, existing studies have provided important scientific evidence for a deeper understanding of the pathological mechanisms of liver cirrhosis and the development of new treatment methods. With more studies on the mechanisms of pyroptosis, more drugs targeting this pathway may be clinically applied in the future.

### 4.7 Limitations and challenges in the clinical application of pyroptosis-related therapies

Pyroptosis-related therapies face a series of limitations and challenges in clinical applications. First, these therapies have difficulty in specificity. How to precisely target the pyroptosis pathway without affecting other cellular processes is an urgent issue to be resolved. Additionally, modulating pyroptosis may interfere with the immune system, leading to immunosuppression and increased risk of infection. Drug delivery is also a challenge. Ensuring that the therapeutic agent effectively reaches the site of disease and achieves an effective concentration is crucial for the therapeutic outcome. Finally, the complexity of diseases means that a single therapy targeting pyroptosis may not comprehensively cover the multifactorial mechanisms of the disease.

## 5 Prospectives

Undoubtedly, pyroptosis plays an important role in liver fibrosis and even cirrhosis; thus, therapy targeting the pyroptosis signaling pathway has promising therapeutic prospects. However, clinical research on small-molecule drug inhibitors or natural plant compounds or nanocarriers for pyroptosis is limited. Therefore, more in-depth basic research and clinical trials are urgently needed. In addition, the molecular mechanisms of pyroptosis mediated by natural plant compounds in liver cirrhosis still have a broad scope for research, and certain molecular mechanisms of pyroptosis may be unrecognized. Finally, to advance the progress of clinical trials, liver organoid simulation of cirrhosis requires in-depth investigation as a pathological model that can maximally recapitulate the pathological and molecular changes of cirrhosis in humans. Specifically, organoid libraries may provide a reliable technology for screening drugs for the pyroptosis-based treatment of cirrhosis.

## 6 Conclusions

As an inflammatory programed death mode, increasing evidence suggests the important role of pyroptosis in liver fibrosis/cirrhosis; however, there are still some issues that need to be urgently addressed, such as how to continuously improve and translate the basic research on pyroptosis and cirrhosis into clinical practice. This study summarizes approaches that mitigate liver fibrosis/cirrhosis through the anti-pyroptosis pathway, including inhibitors targeting NLRP3, GSDMD, and caspases in the pyroptosis pathway, natural plant compounds and biomaterials (SCs and exosomes). These approaches can be effectively targeted to inhibit the pyroptosis pathway in cellular and animal models; however, the specific mechanisms and value in clinical applications must be further investigated. This study provides a reference and a drug list to understand the treatment of liver fibrosis/cirrhosis from a pyroptosis perspective.

## References

[1] Asrani SK, Devarbhavi H, Eaton J, Kamath PS. Burden of liver diseases in the world. J Hepatol. 2019;70(1):151–71. 10.1016/j.jhep.2018.09.014 30266282

[2] Mazumder NR, Celaj S, Atiemo K, Daud A, Jackson KL, Kho A, et al. Liver-related mortality is similar among men and women with cirrhosis. J Hepatol. 2020;73(5):1072–81. 10.1016/j.jhep.2020.04.022 PMC7572539 32344052

[3] Zhang CY, Yuan WG, He P, Lei JH, Wang CX. Liver fibrosis and hepatic stellate cells: Etiology, pathological hallmarks and therapeutic targets. World J Gastroenterol. 2016;22(48):10512–22. 10.3748/wjg.v22.i48.10512 PMC5192262 28082803

[4] Rajapaksha I. Liver fibrosis, liver cancer, and advances in therapeutic approaches. Livers. 2022;2(4):372–86.

[5] Ginès P, Krag A, Abraldes JG, Solà E, Fabrellas N, Kamath PS. Liver cirrhosis. Lancet. 2021;398(10308):1359–76. 10.1016/S0140-6736(21)01374-X 34543610

[6] Vanden Berghe T, Linkermann A, Jouan-Lanhouet S, Walczak H, Vandenabeele P. Regulated necrosis: The expanding network of non-apoptotic cell death pathways. Nat Rev Mol Cell Biol. 2014;15(2):135–47. 10.1038/nrm3737 24452471

[7] Zhao H, Liu H, Yang Y, Wang H. The role of autophagy and pyroptosis in liver disorders. Int J Mol Sci. 2022;23(11):6208. 10.3390/ijms23116208 PMC9181643 35682887

[8] Gaul S, Leszczynska A, Alegre F, Kaufmann B, Johnson CD, Adams LA, et al. Hepatocyte pyroptosis and release of inflammasome particles induce stellate cell activation and liver fibrosis. J Hepatol. 2021;74(1):156–67. 10.1016/j.jhep.2020.07.041 PMC7749849 32763266

[9] Rodriguez-Antonio I, Lopez-Sanchez GN, Uribe M, Chavez-Tapia NC, Nuno-Lambarri N. Role of the inflammasome, gasdermin D, and pyroptosis in non-alcoholic fatty liver disease. J Gastroenterol Hepatol. 2021;36(10):2720–7. 10.1111/jgh.15561 34050551

[10] Alegre F, Pelegrin P, Feldstein AE. Inflammasomes in liver fibrosis. Semin Liver Dis. 2017;37(2):119–27. 10.1055/s-0037-1601350 28564720

[11] Wang J, Sun Z, Xie J, Ji W, Cui Y, Ai Z, et al. Inflammasome and pyroptosis in autoimmune liver diseases. Front Immunol. 2023;14:1150879. 10.3389/fimmu.2023.1150879 PMC10030845 36969233

[12] Strowig T, Henao-Mejia J, Elinav E, Flavell R. Inflammasomes in health and disease. Nature. 2012;481(7381):278–86. 10.1038/nature10759 22258606

[13] Sborgi L, Ruhl S, Mulvihill E, Pipercevic J, Heilig R, Stahlberg H, et al. GSDMD membrane pore formation constitutes the mechanism of pyroptotic cell death. EMBO J. 2016;35(16):1766–78. 10.15252/embj.201694696 PMC5010048 27418190

[14] Wang L, Hauenstein AV. The NLRP3 inflammasome: Mechanism of action, role in disease and therapies. Mol Asp Med. 2020;76:100889. 10.1016/j.mam.2020.100889 32859386

[15] Han Y, Lu Y, Li X, Niu X, Chang AK, Yang Z, et al. Novel organoselenides (NSAIDs-Se derivatives) protect against LPS-induced inflammation in microglia by targeting the NOX2/NLRP3 signaling pathway. Int Immunopharmacol. 2021;101(Pt B):108377. 10.1016/j.intimp.2021.108377 34836795

[16] Xu D, Zhou X, Chen J, Li N, Ruan S, Zuo A, et al. C1q/tumour necrosis factor-related protein-9 aggravates lipopolysaccharide-induced inflammation via promoting NLRP3 inflammasome activation. Int Immunopharmacol. 2022;104:108513. 10.1016/j.intimp.2021.108513 35008006

[17] Zhou R, Tardivel A, Thorens B, Choi I, Tschopp J. Thioredoxin-interacting protein links oxidative stress to inflammasome activation. Nat Immunol. 2010;11(2):136–40. 10.1038/ni.1831 20023662

[18] Yu P, Zhang X, Liu N, Tang L, Peng C, Chen X. Pyroptosis: mechanisms and diseases. Signal Transduct Target Ther. 2021;6(1):128. 10.1038/s41392-021-00507-5 PMC8005494 33776057

[19] Jorgensen I, Miao EA. Pyroptotic cell death defends against intracellular pathogens. Immunol Rev. 2015;265(1):130–42. 10.1111/imr.12287 PMC4400865 25879289

[20] Kayagaki N, Stowe IB, Lee BL, O’Rourke K, Anderson K, Warming S, et al. Caspase-11 cleaves gasdermin D for non-canonical inflammasome signalling. Nature. 2015;526(7575):666–71. 10.1038/nature15541 26375259

[21] Baker PJ, Boucher D, Bierschenk D, Tebartz C, Whitney PG, D’Silva DB, et al. NLRP3 inflammasome activation downstream of cytoplasmic LPS recognition by both caspase-4 and caspase-5. Eur J Immunol. 2015;45(10):2918–26. 10.1002/eji.201545655 26173988

[22] Yang D, He Y, Muñoz-Planillo R, Liu Q, Núñez G. Caspase-11 requires the pannexin-1 channel and the purinergic P2X7 pore to mediate pyroptosis and endotoxic shock. Immunity. 2015;43(5):923–32. 10.1016/j.immuni.2015.10.009 PMC4795157 26572062

[23] Huang C, Yu W, Cui H, Wang Y, Zhang L, Han F, et al. P2X7 blockade attenuates mouse liver fibrosis. Mol Med Rep. 2014;9(1):57–62. 10.3892/mmr.2013.1807 24247209

[24] Liu Y, Fang Y. Gasdermin E-mediated target cell pyroptosis by CAR T cells triggers cytokine release syndrome. Sci Immunol. 2020;5(43):eaax7969. 10.1126/sciimmunol.aax7969 31953257

[25] Zhou Z, He H. Granzyme A from cytotoxic lymphocytes cleaves GSDMB to trigger pyroptosis in target cells. Science. 2020;368(6494). 10.1126/science.aaz7548 32299851

[26] Xia W, Li Y, Wu M, Jin Q, Wang Q, Li S, et al. Gasdermin E deficiency attenuates acute kidney injury by inhibiting pyroptosis and inflammation. Cell Death Dis. 2021;12(2):139. 10.1038/s41419-021-03431-2 PMC7862699 33542198

[27] Rogers C, Fernandes-Alnemri T, Mayes L, Alnemri D, Cingolani G, Alnemri ES. Cleavage of DFNA5 by caspase-3 during apoptosis mediates progression to secondary necrotic/pyroptotic cell death. Nat Commun. 2017;8:14128. 10.1038/ncomms14128 PMC5216131 28045099

[28] Wang Y, Gao W, Shi X, Ding J, Liu W, He H, et al. Chemotherapy drugs induce pyroptosis through caspase-3 cleavage of a gasdermin. Nature. 2017;547(7661):99–103. 10.1038/nature22393 28459430

[29] Orning P, Weng D, Starheim K, Ratner D. Pathogen blockade of TAK1 triggers caspase-8-dependent cleavage of gasdermin D and cell death. Science. 2018;362(6418):1064–9. 10.1126/science.aau2818 PMC6522129 30361383

[30] Sarhan J, Liu BC, Muendlein HI, Li P, Nilson R, Tang AY, et al. Caspase-8 induces cleavage of gasdermin D to elicit pyroptosis during Yersinia infection. Proc Natl Acad Sci U S A. 2018;115(46):E10888–97. 10.1073/pnas.1809548115 PMC6243247 30381458

[31] Wang D, Zhan X, Wu R, You Y, Chen W, Duan L. Assessment of pyroptosis-related indicators as potential biomarkers and their association with severity in patients with liver cirrhosis. J Inflamm Res. 2021;14:3185–96. 10.2147/JIR.S319213 PMC8286130 34285542

[32] Hurtado-Navarro L, Angosto-Bazarra D, Pelegrin P, Baroja-Mazo A, Cuevas S. NLRP3 inflammasome and pyroptosis in liver pathophysiology: The emerging relevance of Nrf2 inducers. Antioxidants (Basel). 2022;11(5):870. 10.3390/antiox11050870 PMC9137763 35624734

[33] Ramos-Tovar E, Muriel P. Molecular mechanisms that link oxidative stress, inflammation, and fibrosis in the liver. Antioxidants (Basel). 2020;9(12):1279. 10.3390/antiox9121279 PMC7765317 33333846

[34] Wan Y, Zhang W, Huang C, Jian J, Zhang Y, Liu Q, et al. Ursolic acid alleviates Kupffer cells pyroptosis in liver fibrosis by the NOX2/NLRP3 inflammasome signaling pathway. Int Immunopharmacol. 2022;113(Pt A):109321. 10.1016/j.intimp.2022.109321 36252479

[35] Li T, Yang Y, Song H, Li H, Cui A, Liu Y, et al. Activated NK cells kill hepatic stellate cells via p38/PI3K signaling in a TRAIL-involved degranulation manner. J Leukoc Biol. 2019;105(4):695–704. 10.1002/JLB.2A0118-031RR 30748035

[36] Kong DL, Kong FY, Liu XY, Yan C, Cui J, Tang RX, et al. Soluble egg antigen of Schistosoma japonicum induces pyroptosis in hepatic stellate cells by modulating ROS production. Parasit Vectors. 2019;12(1):475. 10.1186/s13071-019-3729-8 PMC6791022 31610797

[37] Yuan J, Najafov A, Py BF. Roles of caspases in necrotic cell death. Cell. 2016;167(7):1693–704. 10.1016/j.cell.2016.11.047 PMC5381727 27984721

[38] Christgen S, Place DE, Kanneganti TD. Toward targeting inflammasomes: insights into their regulation and activation. Cell Res. 2020;30(4):315–27. 10.1038/s41422-020-0295-8 PMC7118104 32152420

[39] Gan C, Cai Q, Tang C, Gao J. Inflammasomes and pyroptosis of liver cells in liver fibrosis. Front Immunol. 2022;13:896473. 10.3389/fimmu.2022.896473 PMC9189314 35707547

[40] Koh EH, Yoon JE, Ko MS, Leem J, Yun JY, Hong CH, et al. Sphingomyelin synthase 1 mediates hepatocyte pyroptosis to trigger non-alcoholic steatohepatitis. Gut. 2021;70(10):1954–64. 10.1136/gutjnl-2020-322509 PMC8458090 33208407

[41] Coll RC, Robertson AA, Chae JJ, Higgins SC, Munoz-Planillo R, Inserra MC, et al. A small-molecule inhibitor of the NLRP3 inflammasome for the treatment of inflammatory diseases. Nat Med. 2015;21(3):248–55. 10.1038/nm.3806 PMC4392179 25686105

[42] Qu J, Yuan Z, Wang G, Wang X, Li K. The selective NLRP3 inflammasome inhibitor MCC950 alleviates cholestatic liver injury and fibrosis in mice. Int Immunopharmacol. 2019;70:147–55. 10.1016/j.intimp.2019.02.016 30802677

[43] Jiao Y, Nan J, Mu B, Zhang Y, Zhou N, Yang S, et al. Discovery of a novel and potent inhibitor with differential species-specific effects against NLRP3 and AIM2 inflammasome-dependent pyroptosis. Eur J Med Chem. 2022;232:114194. 10.1016/j.ejmech.2022.114194 35183871

[44] Ross C, Chan AH, von Pein JB, Maddugoda MP, Boucher D, Schroder K. Inflammatory caspases: Toward a unified model for caspase activation by inflammasomes. Annu Rev Immunol. 2022;40:249–69. 10.1146/annurev-immunol-101220-030653 35080918

[45] Yang H, Wang J, Liu ZG. Multi-faceted role of pyroptosis mediated by inflammasome in liver fibrosis. J Cell Mol Med. 2022;26(10):2757–65. 10.1111/jcmm.17277 PMC9097829 35415891

[46] Witek RP, Stone WC, Karaca FG, Syn WK, Pereira TA, Agboola KM, et al. Pan-caspase inhibitor VX-166 reduces fibrosis in an animal model of nonalcoholic steatohepatitis. Hepatology. 2009;50(5):1421–30. 10.1002/hep.23167 19676126

[47] Barreyro FJ, Holod S, Finocchietto PV, Camino AM, Aquino JB, Avagnina A, et al. The pan-caspase inhibitor Emricasan (IDN-6556) decreases liver injury and fibrosis in a murine model of non-alcoholic steatohepatitis. Liver Int. 2015;35(3):953–66. 10.1111/liv.12570 24750664

[48] Morrison MC, Mulder P, Salic K, Verheij J, Liang W, van Duyvenvoorde W, et al. Intervention with a caspase-1 inhibitor reduces obesity-associated hyperinsulinemia, non-alcoholic steatohepatitis and hepatic fibrosis in LDLR-/-.Leiden mice. Int J Obes (Lond). 2016;40(9):1416–23. 10.1038/ijo.2016.74 PMC5022108 27121255

[49] Zhu Y, Zhao H, Lu J, Lin K, Ni J, Wu G, et al. Caspase-11-mediated hepatocytic pyroptosis promotes the progression of nonalcoholic steatohepatitis. Cell Mol Gastroenterol Hepatol. 2021;12(2):653–64. 10.1016/j.jcmgh.2021.04.009 PMC8261017 33894425

[50] Shi J, Zhao Y, Wang K, Shi X, Wang Y, Huang H, et al. Cleavage of GSDMD by inflammatory caspases determines pyroptotic cell death. Nature. 2015;526(7575):660–5. 10.1038/nature15514 26375003

[51] He WT, Wan H, Hu L, Chen P, Wang X, Huang Z, et al. Gasdermin D is an executor of pyroptosis and required for interleukin-1beta secretion. Cell Res. 2015;25(12):1285–98. 10.1038/cr.2015.139 PMC4670995 26611636

[52] Zhang T, Li J, Liu CP, Guo M, Gao CL, Zhou LP, et al. Butyrate ameliorates alcoholic fatty liver disease via reducing endotoxemia and inhibiting liver gasdermin D-mediated pyroptosis. Ann Transl Med. 2021;9(10):873. 10.21037/atm-21-2158 PMC8184481 34164507

[53] Ding J, Wang K, Liu W, She Y, Sun Q, Shi J, et al. Pore-forming activity and structural autoinhibition of the gasdermin family. Nature. 2016;535(7610):111–6. 10.1038/nature18590 27281216

[54] Hu JJ, Liu X, Xia S, Zhang Z, Zhang Y, Zhao J, et al. FDA-approved disulfiram inhibits pyroptosis by blocking gasdermin D pore formation. Nat Immunol. 2020;21(7):736–45. 10.1038/s41590-020-0669-6 PMC7316630 32367036

[55] Deng W, Yang Z, Yue H, Ou Y, Hu W, Sun P. Disulfiram suppresses NLRP3 inflammasome activation to treat peritoneal and gouty inflammation. Free Radic Biol Med. 2020;152:8–17. 10.1016/j.freeradbiomed.2020.03.007 32151746

[56] Yin K, Zhou X, Jiang W, Wang L, Dai Z, Tang B. Jiangzhi Ligan Decoction inhibits GSDMD-mediated canonical/noncanonical pyroptosis pathways and alleviates high-fat diet-induced nonalcoholic fatty liver disease. Dis Markers. 2021;2021:9963534. 10.1155/2021/9963534 PMC8235964 34239622

[57] Tang B. Action mechanism of Jiangzhi Ligan Decoction in treatment of non-alcoholic fatty liver disease based on network pharmacology. Chin Tradit Herb Drugs. 2018;49(15):3493–500.

[58] Zhao X, Wang J, Deng Y, Liao L, Zhou M, Peng C, et al. Quercetin as a protective agent for liver diseases: A comprehensive descriptive review of the molecular mechanism. Phytother Res. 2021;35(9):4727–47. 10.1002/ptr.7104 34159683

[59] Kai J, Yang X, Wang Z, Wang F, Jia Y, Wang S, et al. Oroxylin a promotes PGC-1alpha/Mfn2 signaling to attenuate hepatocyte pyroptosis via blocking mitochondrial ROS in alcoholic liver disease. Free Radic Biol Med. 2020;153:89–102. 10.1016/j.freeradbiomed.2020.03.031 32289481

[60] Zhao X, Wang C, Dai S, Liu Y, Zhang F, Peng C, et al. Quercetin protects ethanol-induced hepatocyte pyroptosis via scavenging mitochondrial ROS and promoting PGC-1alpha-regulated mitochondrial homeostasis in L02 cells. Oxid Med Cell Longev. 2022;2022:4591134. 10.1155/2022/4591134 PMC9308520 35879991

[61] Na L, Zhang Q, Jiang S, Du S, Zhang W, Li Y, et al. Mangiferin supplementation improves serum lipid profiles in overweight patients with hyperlipidemia: a double-blind randomized controlled trial. Sci Rep. 2015;5:10344. 10.1038/srep10344 PMC4437311 25989216

[62] Cargnin ST, Gnoatto SB. Ursolic acid from apple pomace and traditional plants: A valuable triterpenoid with functional properties. Food Chem. 2017;220:477–89. 10.1016/j.foodchem.2016.10.029 27855928

[63] Zhu X, Bian H, Gao X. The potential mechanisms of berberine in the treatment of nonalcoholic fatty liver disease. Molecules. 2016;21(10):1336. 10.3390/molecules21101336 PMC6273247 27754444

[64] Mai W, Xu Y, Xu J, Zhao D, Ye L, Yu G, et al. Berberine inhibits nod-like receptor family pyrin domain containing 3 inflammasome activation and pyroptosis in nonalcoholic steatohepatitis via the ROS/TXNIP axis. Front Pharmacol. 2020;11:185. 10.3389/fphar.2020.00185 PMC7063468 32194416

[65] Zhang J, Chen Y, Luo H, Sun L, Xu M, Yu J, et al. Recent update on the pharmacological effects and mechanisms of dihydromyricetin. Front Pharmacol. 2018;9:1204. 10.3389/fphar.2018.01204 PMC6209623 30410442

[66] Cheng QC, Fan J, Deng XW, Liu HC, Ding HR, Fang X, et al. Dihydromyricetin ameliorates chronic liver injury by reducing pyroptosis. World J Gastroenterol. 2020;26(41):6346–60. 10.3748/wjg.v26.i41.6346 PMC7656208 33244197

[67] Peng Z, Li X, Xing D, Du X, Wang Z, Liu G, et al. Nobiletin alleviates palmitic acid‑induced NLRP3 inflammasome activation in a sirtuin 1‑dependent manner in AML‑12 cells. Mol Med Rep. 2018;18(6):5815–22. 10.3892/mmr.2018.9615 30387829

[68] Hernandez-Aquino E, Muriel P. Beneficial effects of naringenin in liver diseases: Molecular mechanisms. World J Gastroenterol. 2018;24(16):1679–707. 10.3748/wjg.v24.i16.1679 PMC5922990 29713125

[69] Wang Y, Mukhopadhyay P, Cao Z, Wang H, Feng D, Hasko G, et al. Cannabidiol attenuates alcohol-induced liver steatosis, metabolic dysregulation, inflammation and neutrophil-mediated injury. Sci Rep. 2017;7(1):12064. 10.1038/s41598-017-10924-8 PMC5608708 28935932

[70] Huang Y, Wan T, Pang N, Zhou Y, Jiang X, Li B, et al. Cannabidiol protects livers against nonalcoholic steatohepatitis induced by high-fat high cholesterol diet via regulating NF-kappaB and NLRP3 inflammasome pathway. J Cell Physiol. 2019;234(11):21224–34. 10.1002/jcp.28728 31032942

[71] Jiang X, Gu Y, Huang Y, Zhou Y, Pang N, Luo J, et al. CBD alleviates liver injuries in alcoholics with high-fat high-cholesterol diet through regulating NLRP3 inflammasome-pyroptosis pathway. Front Pharmacol. 2021;12:724747. 10.3389/fphar.2021.724747 PMC8493333 34630100

[72] Panossian A, Wagner H. Stimulating effect of adaptogens: An overview with particular reference to their efficacy following single dose administration. Phytother Res. 2005;19(10):819–38. 10.1002/ptr.1751 16261511

[73] Zheng T, Yang X, Li W, Wang Q, Chen L, Wu D, et al. Salidroside attenuates high-fat diet-induced nonalcoholic fatty liver disease via AMPK-dependent TXNIP/NLRP3 pathway. Oxid Med Cell Longev. 2018;2018:8597897. 10.1155/2018/8597897 PMC6081551 30140371

[74] Oh S, Son M, Byun KA, Jang JT, Choi CH, Son KH, et al. Attenuating effects of dieckol on high-fat diet-induced nonalcoholic fatty liver disease by decreasing the NLRP3 inflammasome and pyroptosis. Mar Drugs. 2021;19(6):318. 10.3390/md19060318 PMC8227003 34070893

[75] Ruan S, Han C, Sheng Y, Wang J, Zhou X, Guan Q, et al. Antcin A alleviates pyroptosis and inflammatory response in Kupffercells of non-alcoholic fatty liver disease by targeting NLRP3. Int Immunopharmacol. 2021;100:108126. 10.1016/j.intimp.2021.108126 34492534

[76] Zeng X, Zhu M, Liu X, Chen X, Yuan Y, Li L, et al. Oleic acid ameliorates palmitic acid induced hepatocellular lipotoxicity by inhibition of ER stress and pyroptosis. Nutr Metab (Lond). 2020;17:11. 10.1186/s12986-020-0434-8 PMC6990600 32021639

[77] Qiu T, Pei P, Yao X, Jiang L, Wei S, Wang Z, et al. Taurine attenuates arsenic-induced pyroptosis and nonalcoholic steatohepatitis by inhibiting the autophagic-inflammasomal pathway. Cell Death Dis. 2018;9(10):946. 10.1038/s41419-018-1004-0 PMC6148242 30237538

[78] Budanov AV. Stress-responsive sestrins link p53 with redox regulation and mammalian target of rapamycin signaling. Antioxid Redox Signal. 2011;15(6):1679–90. 10.1089/ars.2010.3530 PMC3151419 20712410

[79] Han D, Kim H, Kim S, Le QA, Han SY, Bae J, et al. Sestrin2 protects against cholestatic liver injury by inhibiting endoplasmic reticulum stress and NLRP3 inflammasome-mediated pyroptosis. Exp Mol Med. 2022;54(3):239–51. 10.1038/s12276-022-00737-9 PMC8980001 35260799

[80] Wang Y, Zhao Y, Wang Z, Sun R, Zou B, Li R, et al. Peroxiredoxin 3 inhibits acetaminophen-induced liver pyroptosis through the regulation of mitochondrial ROS. Front Immunol. 2021;12:652782. 10.3389/fimmu.2021.652782 PMC8155593 34054813

[81] Cui Y, Wang Q, Zhang X, Yang X, Shi Y, Li Y, et al. Curcumin alleviates aflatoxin B(1)-induced liver pyroptosis and fibrosis by regulating the JAK2/NLRP3 signaling pathway in ducks. Foods. 2023;12(5):1006. 10.3390/foods12051006 PMC10000391 36900523

[82] Petersen BE, Bowen WC, Patrene KD, Mars WM, Sullivan AK, Murase N, et al. Bone marrow as a potential source of hepatic oval cells. Science. 1999;284(5417):1168–70. 10.1126/science.284.5417.1168 10325227

[83] Chen P, Zhou YK, Han CS, Chen LJ, Wang YM, Zhuang ZM, et al. Stem cells from human exfoliated deciduous teeth alleviate liver cirrhosis via inhibition of gasdermin D-executed hepatocyte pyroptosis. Front Immunol. 2022;13:860225. 10.3389/fimmu.2022.860225 PMC9133376 35634294

[84] Liu M, He J, Zheng S, Zhang K, Ouyang Y, Zhang Y, et al. Human umbilical cord mesenchymal stem cells ameliorate acute liver failure by inhibiting apoptosis, inflammation and pyroptosis. Ann Transl Med. 2021;9(21):1615. 10.21037/atm-21-2885 PMC8640895 34926659

[85] Wu H-W, Chen H-D, Chen Y-H, Mao X-L, Feng Y-Y, Li S-W, et al. The effects of programmed cell death of mesenchymal stem cells on the development of liver fibrosis. Stem Cell Int. 2023;2023:4586398. 10.1155/2023/4586398 PMC10195177 37214784

[86] Sattwika PD, Indrarti F, Bayupurnama P. Clinical application of stem cell therapy for liver cirrhosis: Progress, pitfalls, and prospects. Acta Med Indones. 2021;53(4):473–80. 35027497

[87] He C, Zheng S, Luo Y, Wang B. Exosome theranostics: Biology and translational medicine. Theranostics. 2018;8(1):237–55. 10.7150/thno.21945 PMC5743472 29290805

[88] Zhang Y, Zhangdi H, Nie X, Wang L, Wan Z, Jin H, et al. Exosomes derived from BMMSCs mitigate the hepatic fibrosis via anti-pyroptosis pathway in a cirrhosis model. Cells. 2022;11(24):4004. 10.3390/cells11244004 PMC9776579 36552767

[89] Gong L, Zhou H, Zhang S, Wang C, Fu K, Ma C, et al. CD44-targeting drug delivery system of exosomes loading forsythiaside a combats liver fibrosis via regulating NLRP3-mediated pyroptosis. Adv Healthc Mater. 2023;12(11):e2202228. 10.1002/adhm.202202228 36603210

[90] Tan F, Li X, Wang Z, Li J, Shahzad K, Zheng J. Clinical applications of stem cell-derived exosomes. Signal Transduct Target Ther. 2024;9(1):17. 10.1038/s41392-023-01704-0 PMC10784577 38212307

[91] Wang C, Liu J, Yan Y, Tan Y. Role of exosomes in chronic liver disease development and their potential clinical applications. J Immunol Res. 2022;2022:1695802. 10.1155/2022/1695802 PMC9106457 35571570

[92] Cook D, Brown D, Alexander R, March R, Morgan P, Satterthwaite G, et al. Lessons learned from the fate of AstraZeneca’s drug pipeline: A five-dimensional framework. Nat Rev Drug Discov. 2014;13(6):419–31. 10.1038/nrd4309 24833294

[93] Liu J, MacNaughtan J, Kerbert AJC, Portlock T, Martinez Gonzalez J, Jin Y, et al. Clinical, experimental and pathophysiological effects of Yaq-001: A non-absorbable, gut-restricted adsorbent in models and patients with cirrhosis. Gut. 2024;73(7):1183–98. 10.1136/gutjnl-2023-330699 38621924

[94] Wen J, Chen X, Wei S, Ma X, Zhao Y. Research progress and treatment status of liver cirrhosis with hypoproteinemia. Evid Based Complement Altern Med. 2022;2022:2245491. 10.1155/2022/2245491 PMC8893996 35251204

[95] Fernandez J, Piano S, Bartoletti M, Wey EQ. Management of bacterial and fungal infections in cirrhosis: The MDRO challenge. J Hepatol. 2021;75(Suppl 1):S101–17. 10.1016/j.jhep.2020.11.010 34039482

[96] Zhang L, Deng Y, Bai X, Wei X, Ren Y, Chen S, et al. Cell therapy for end-stage liver disease: Current state and clinical challenge. Chin Med J (Engl). 2024;137(23):2808–20. 10.1097/CM9.0000000000003332 PMC11649288 39602326

[97] Wong GL, Chan HL, Mak CW, Lee SK, Ip ZM, Lam AT, et al. Entecavir treatment reduces hepatic events and deaths in chronic hepatitis B patients with liver cirrhosis. Hepatology. 2013;58(5):1537–47. 10.1002/hep.26301 23389810

[98] Zeng X, Sheng X, Wang PQ, Xin HG, Guo YB, Lin Y, et al. Low-dose rifaximin prevents complications and improves survival in patients with decompensated liver cirrhosis. Hepatol Int. 2021;15(1):155–65. 10.1007/s12072-020-10117-y 33385299

[99] Brennan PN, MacMillan M, Manship T, Moroni F, Glover A, Troland D, et al. Autologous macrophage therapy for liver cirrhosis: A phase 2 open-label randomized controlled trial. Nat Med. 2025;31(3):979–87. 10.1038/s41591-024-03406-8 PMC11922741 39794616

[100] Rinella ME, Lieu HD, Kowdley KV, Goodman ZD, Alkhouri N, Lawitz E, et al. A randomized, double-blind, placebo-controlled trial of aldafermin in patients with NASH and compensated cirrhosis. Hepatology. 2024;79(3):674–89. 10.1097/HEP.0000000000000607 PMC10871650 37732990

